# Formulation and characterization of cholesterol-based nanoparticles of gabapentin protecting from retinal injury

**DOI:** 10.3389/fchem.2024.1449380

**Published:** 2024-10-21

**Authors:** Hatem I. Mokhtar, Dina M. Khodeer, Sharifa Alzahrani, Mona Qushawy, Reem Alshaman, Nehal M. Elsherbiny, Esam Sayed Ahmed, Esam Ghanem Abu El Wafa, Mohamed K. El-Kherbetawy, Ahmed R. Gardouh, Sawsan A. Zaitone

**Affiliations:** ^1^ Department of Pharmaceutical Chemistry, Faculty of Pharmacy, Sinai University-Kantara Branch, Ismailia, Egypt; ^2^ Department of Pharmacology and Toxicology, Faculty of Pharmacy, Suez Canal University, Ismailia, Egypt; ^3^ Department of Pharmacology, Faculty of Medicine, University of Tabuk, Tabuk, Saudi Arabia; ^4^ Department of Pharmaceutics, Faculty of Pharmacy, University of Tabuk, Tabuk, Saudi Arabia; ^5^ Department of Pharmacology and Toxicology, Faculty of Pharmacy, University of Tabuk, Tabuk, Saudi Arabia; ^6^ Department of Pharmaceutical Chemistry, Faculty of Pharmacy, University of Tabuk, Tabuk, Saudi Arabia; ^7^ Department of Ophthalmology, Al-Azher Asyut Faculty of Medicine for Men, Asyut, Egypt; ^8^ Department of Pathology, Faculty of Medicine, Suez Canal University, Ismailia, Egypt; ^9^ Department of Pharmaceutics and Industrial Pharmacy, Faculty of Pharmacy, Suez Canal University, Ismailia, Egypt; ^10^ Department of Pharmaceutical Sciences, Faculty of Pharmacy, Jadara University, Irbid, Jordan

**Keywords:** gabapentin, IL6/JAK2/STAT3 signaling, retinal injury, rat, solid lipid nanoparticles

## Abstract

**Introduction:**

This study aimed to prepare cholesterol and stearic acid-based solid lipid nanoparticles of gabapentin (GAB-SLNs) for protection against streptozotocin (STZ)-induced retinal injury in rats.

**Methods:**

We prepared four preparations of GAB-SLNs using a hot high-shear homogenization ultrasonication process, and the best formulation was selected and tested for biological activity. The retinal injury was brought in male adult albino rats while gabapentin doses continued for 6 weeks. Six groups of rats were assigned as the vehicle, diabetic, diabetic + gabapentin (10–20 mg/kg), and diabetic + GAB-SLNs (10–20 mg/kg). GAB-SLN#2 was selected as the optimized formulation with high entrapment efficacy (EE%, 98.64% ± 1.97%), small particle size (185.65 ± 2.41 nm), high negative Zeta potential (−32.18 ± 0.98 mV), low polydispersity index (0.28 ± 0.02), and elevated drug release (99.27% ± 3.48%). The TEM image of GAB-SLN#2 revealed a smooth surface with a spherical shape.

**Results:**

GAB-SLNs provided greater protection against retinal injury than free gabapentin as indicated by the histopathology data which demonstrated more organization of retinal layers and less degeneration in ganglion cell layer in rats treated with GAB-SLN#2. Further, GAB-SLN#2 reduced the inflammatory proteins (IL-6/JAK2/STAT3) and vascular endothelial growth factor (VEGF).

**Conclusion:**

The preparation of GAB-SLNs enhanced the physical properties of gabapentin and improved its biological activity as a neuroprotectant. Further studies are warranted to validate this technique for the use of oral gabapentin in other neurological disorders.

## 1 Introduction

Diabetic retinal injury (DR) is a prevalent cause of acquired blindness worldwide (Tomita et al., 2020). It is a neurovascular complication affecting about one-third of diabetic patients. Of note, 10% of DR patients experience severe visual impairment (Yang et al., 2020). Additionally, despite regular and effective screening and early treatment, DR-induced vision loss manifests in people of working age (Fogli et al., 2018). The clinical features of DR include disrupted retinal blood vessels, vascular occlusion, and ischemia with subsequent sight-threatening neovascularization and hemorrhage (Nentwich and Ulbig, 2015). Hence, the current therapeutic approaches use laser therapy, intravitreal steroids, and anti-vascular endothelial growth factor (VEGF) drugs to control changes in the retina’s vascular structure. Despite the remarkable effects of advent DR therapies, the majority of DR patients do not have noticeable improvements in their visual functions, and treatment of DR is still challenging (Wang et al., 2020). In fact, the current therapies are usually administered at rather advanced stages and may impact retinal vascular homeostasis. Consequently, there is a strong need to find alternative interventions for the treatment of DR (Kim et al., 2020).

Gabapentin is a γ-aminobutyric acid analogue initially used as anticonvulsant (Yosri et al., 2018). Laterally, a growing body of evidence reported additional pharmacological uses of gabapentin for the reduction of neuropathic pain, post-operative pain, and allodynia. These therapeutic uses are attributed to its blocking effect on voltage-gated calcium ion channels (α2δ subtype). Additionally, gabapentin through pleiotropic effects, possesses anti-inflammatory effects (de Brito et al., 2020). In this context, gabapentin has been demonstrated to suppress pro-inflammatory cytokines in an experimental model of neuropathic pain (Lee et al., 2013). Moreover, it counteracted inflammatory conditions in acute mouse models of inflammation (Dias et al., 2014). Interestingly, anti-inflammatory effects were documented for the ophthalmic formulation of gabapentin in experimentally-induced uveitis (Anfuso et al., 2017) and topical formulations to treat ocular surface diseases (Rusciano, 2024). Moreover, the anti-oxidant and anti-apoptotic effects of gabapentin were shown previously in diabetic rat retinas and this effect was explained based on inhibiting glutamate excitotoxicity (Ola et al., 2019).

Enhancing drug bioavailability and pharmacokinetic properties via nano-drug delivery systems has been getting much attention (Patra et al., 2018; Abdellatif et al., 2023; Gopu et al., 2023). Due to its hydrophobicity, the oral bioavailability of gabapentin is low. Indeed, its gastrointestinal absorption occurs via a capacity capacity-limited transport system. Therefore, its plasma concentration does not increase proportionally with the dose ingested. Indeed, gabapentin shows a saturable absorption pathway, zero-order absorption pharmacokinetic, and absolute bioavailability that decreases from 60% to 33% with increasing the daily oral dose from 900 to 3,600 mg (Bockbrader et al., 2010). These properties necessitate the administration of gabapentin three times daily which causes patients inconvenience and increases the probability of side effects. Fortunately, this can be probably minimized via nanoformulation of gabapentin which may help in reducing the daily dose and allowing slow release of the drug in blood to provide steady blood concentration over a longer period hence enhancing its therapeutic efficacy (Wilson et al., 2014).

Solid lipid nanoparticles (SLNs) are spherical platforms with diameters ranging from 50 to 1,000 nm. SLNs are a new type of lipid emulsions that are smaller than 1 micron in which a high melting point solid lipid is used instead of oil (Geszke-Moritz and Moritz, 2016). Indeed, the use of these colloidal dispersions allows the successful overcoming of major problems encountered with other colloidal nanoparticle preparations such as drug leakage, polymer breakdown and cytotoxicity, the absence of an appropriate large-scale production process, the high production cost, and challenges with sterilization (QUSHAWY and Nasr, 2020; Scioli Montoto et al., 2020). Because of their tiny size, broad surface area, flexible dosing forms, and enhanced loading capacity, SLNs are currently gaining a great deal of attention from formulators worldwide. This is done to improve the performance and bioavailability of pharmaceuticals. In addition, SLNs can both facilitate the regulated release of the entrapped medication and improve the stability of the drug that has been entrapped. The trapped drug is dispersed or dissolved within the hydrophobic core of the SLNs and is enveloped by a monolayer covering composed of phospholipids. The prospects of lipid-based drug delivery systems are largely contingent upon SLNs (Mishra et al., 2018). Gabapentin is a hydrophilic drug with low bioavailability, so preparation as SLNs is expected to improve the drug permeation through the biological membranes and hence, increase the bioavailability. Further, SLNs control the drug release which helps reduce the number of daily doses and improves patient compliance.

Gabapentin is a hydrophilic drug that belongs to BCS class III which is characterized by high water solubility and low permeability. To improve the permeability, it is beneficial to entrap the drug in a lipid matrix to facilitate the permeation. SLNs are a colloidal dispersion of non-polar lipids such as triglycerides and fatty acids, which are solid at room temperature as well as at the body temperature. Since the basic template of SLNs is a solid lipid, their affinity towards lipophilic drugs is higher when compared to hydrophilic drugs (Dave et al., 2017; Mallick et al., 2020).

Given the potential neuroprotective efficacy of gabapentin in neurologic disorders and the several advantages associated with the use of SLNs, this study aimed to prepare novel preparations of cholesterol and stearic acid-based GAB-SLNs and select the best formulation to be investigated for its neuroprotective effect and impact on IL-6/JAK2/STAT3 signaling in experimental retinal injury in comparison to a free gabapentin preparation.

## 2 Materials and methods

### 2.1 Formulation of cholesterol and stearic acid-based solid-lipid nanoparticles of gabapentin

#### 2.1.1 Drugs and chemicals

The gabapentin powder used in this study was a gift from Eva Pharmaceutical Company (Cairo, Egypt). Cholesterol was obtained from SDFCL, a company (Mumbai, India). Stearic acid was procured from Hi-Media Laboratories Pvt. Ltd (Mumbai, India). Pluronic F-68 was acquired from Sigma Aldrich (MO, USA).

#### 2.1.2 Application of 2^2^ factorial designs to formulate GAB-SLNs

One of the optimization methods is a 2-level 2-factor design. This design results in four different formulations. Many research used the same design for the optimization process (Qushawy et al., 2022; Alshaman et al., 2023). The factorial design holds significant importance within the field of pharmaceutical preparation (Zielińska et al., 2018). The present work employed a 2^2^-factorial design to develop and optimize formulations of GAB-SLNs. The study employed two distinct independent variables, namely, the solid lipid (A; X1) and the surfactant (B; X2), each one was used at two different levels. The study focused on examining the impact of independent variables on five dependent variables, including entrapment efficiency % (EE%) as Y1, particle size (PS) Y2, zeta potential (ZP) Y3, polydispersity index (PDI) as Y4, and the % of released drug after 12 h (R) Y5. In Table 1, the independent variables and dependent variables are recorded.

**TABLE 1:** The variables of 2^2^ factorial design for GAB-SLNs.

Independent variables	Name	Level used	
		Low (-1)	High (+1)
A: X1	The lipid	Stearic acid	Cholesterol
B: X2	The surfactant	Tween 80	Pluronic F-68

Dependent variables (Responses)	Name	Target	
Y1	Entrapment Efficacy %	Maximize	
Y2	Particle Size (nm)	Minimize	
Y3	Zeta Potential (mV)	Maximize	
Y4	PDI	Minimize	
Y5	R (%)	Maximize	

#### 2.1.3 Preparation of GAB-SLNs

The modified hot high-shear homogenization ultrasonication process was used to develop four GAB-SLN formulations (El-Housiny et al., 2018). In a small glass vial, an accurate weight of solid lipid (100 g of stearic acid or cholesterol) was added and heated at 80 °C (which is greater than the melting point of the two lipid constituents) utilizing a hot plate (Brandstead/Thermolyne, USA). We prepared the aqueous phase by dissolving gabapentin (10 mg) and surfactant (1% of tween 80 or Pluronic F-68) in distilled water (20 mL) under heating. To make the coarse emulsion O/W, we added the aqueous phase in a slow pattern to the lipid phase, and homogenization of the mixture was performed with a Heidolph silent crusher^®^ homogenizer (Germany) for 11 min at 19,000 rpm (Mohammadi et al., 2020). By sonicating the coarse emulsion with a digital sonifier (Branson, Danbury, USA) for 5 minutes at its maximum output, the coarse emulsion was refined into a fine emulsion (Dudhipala et al., 2020). The sonicated mixture was left at room temperature and allowed to solidify. and then was kept at 4°C (Campos et al., 2019). Table 2 demonstrates the composition of GAB-SLNs.

**TABLE 2:** The designed formulation of GAB-SLNs.

Formula No.	GAB (mg)	The lipid	The surfactant
GAB-SLN #1	10	Cholesterol	Tween 80
GAB-SLN #2	10	Cholesterol	Pluronic F-68
GAB-SLN #3	10	Stearic acid	Tween 80
GAB-SLN #4	10	Stearic acid	Pluronic F-68

GAB: gabapentin, SLNs: solid lipid nanoparticles.

#### 2.1.4 Evaluation of GAB-SLNs in terms of the EE% (Y1)

The indirect determination of the EE% of gabapentin in the produced GAB-SLNs was conducted by applying the centrifugation method, as described in the earlier study (Qushawy et al., 2019). A small volume of each formulation (2 mL) was subjected to centrifugation at a speed of 15,000 rpm for half an hour to facilitate the separation of the entrapped gabapentin from the un-entrapped portion. The transparent liquid portion that included the unentrapped drug was examined using spectrophotometric analysis to determine the presence of unentrapped gabapentin. This analysis was conducted utilizing a UV spectrophotometer (Shimadzu, Japan), with a wavelength of 198 nm. The equation presented below was applied to calculate the EE%:

$$\left(\text{EE}\%\right)=\frac{\text{Total}\;\text{GAB}-\text{Unentrapped}\;\text{GAB}}{\text{Total}\;\text{GAB}}\times 100$$

**FIGURE 1:**
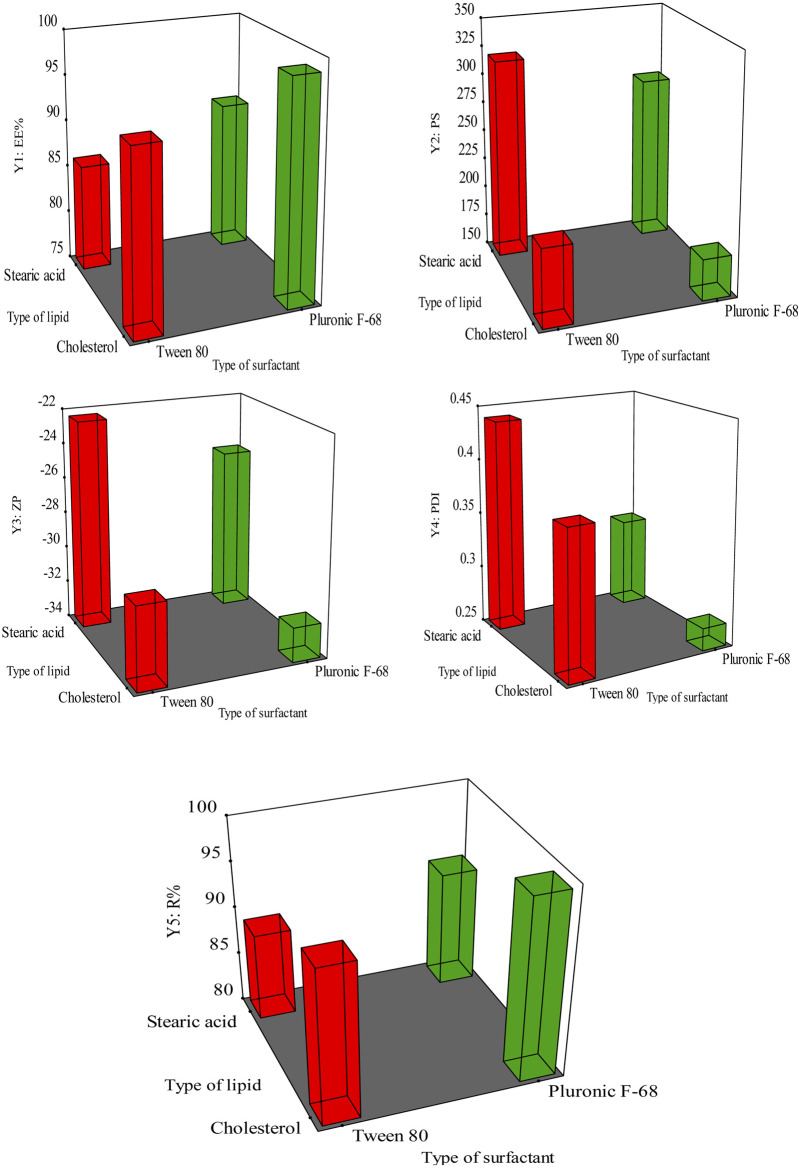
The response surface methodology demonstrating the impact of X1, and X2 on (Y1) EE% (Y2) PS, (Y3) ZP (Y4) PDI, and (Y5) R%.

Spectrophotometric measurements of unentrapped gabapentin for indirect determination of %EE were performed at a wavelength of 198 nm. Gabapentin UV maximum absorbance wavelength ranges from less than 190–206 nm according to the solvent system applied in measurement [reference]. The applied wavelength value at 198 nm represented the maximum absorbance of the gabapentin spectrum in formulation centrifugate in comparison to gabapentin-free blank preparation obtained from experiments (Fonseca et al., 2017). The calibration curve for gabapentin was done in a phosphate buffer pH of 7.4. The linearity was in concentration 10–100 mcg/mL using phosphate buffer as a blank (Supplementary Table S1; Figure 1).

#### 2.1.5 Evaluation of GAB-SLNs

The study involved the evaluation of four different formulations of GAB-SLNs in terms of PS (Y2), ZP (Y3), and PDI (Y4). Additionally, the influence of various formulation parameters on these responses was examined. The GAB-SLNs samples were diluted to a concentration of 1% and subjected to analysis using the Malvern Zetasizer instrument (Malvern, United Kingdom). We applied the dynamic light scattering technique at an angle of 90°C and 25°C (Zielińska et al., 2019; Talarico et al., 2021).

#### 2.1.6 The *in vitro* release experiment (Y5: R) of GAB-SLNs

The Franz’s diffusion cell apparatus (Mumbai, India) was used to perform *in vitro* release studies of the GAB-SLNs formulations (Khan A. S. et al., 2022). The donor compartment received an exact volume of each formulation (2 mL), and 20 mL of phosphate buffer (pH of 7.4) was loaded to the receptor compartment. We stirred the dissolution media at 90 rpm at 37°C. A cellophane membrane with a diameter of 1.7 cm was positioned between the two compartments. Samples were taken from the compartment at 1, 2, 4, 6, 8, 10, and 12 h. Each sample was subjected to spectrophotometric analysis at 198 nm. We carried out this experiment trice to confirm accurate results and then calculated their average. To determine the ideal mechanism, including the order or model, of drug release, a mathematical analysis was done on the drug release data (Ramesh and Mandal, 2019). This analysis was carried out to determine the optimal mechanism.

#### 2.1.7 The GAB-SLNs optimization procedure

The optimal formulation of GAB-SLNs was selected based on (Y1) the maximum EE % (Y2) minimum PS, (Y3) maximum ZP (Y4) minimum PDI, and (Y5) maximum R%. 2^2^ factorial designs, specifically utilizing Design-Expert 11 software, assessed the identification of the optimal values for the independent variables (X1 and X2) to produce GAB-SLNs acquiring the desired response.

#### 2.1.8 Transmission electron microscopy of the optimal GAB-SLNs

The morphology of the GAB-SLNs optimized formula was determined through the use of a JEOL^®^ transmission electron microscope (TEM) (Tokyo, Japan) (Makoni et al., 2019; Khan Z. U. et al., 2022). After adequate dilution with distilled water, a single droplet of the optimized formulation was carefully deposited onto a copper grid coated with collodion (Rodenak-Kladniew et al., 2017). Following air-drying, the sample was subjected to staining using a solution of uranyl acetate. Subsequently, the stained sample was imaged using TEM (Arantes et al., 2020).

#### 2.1.9 The fourier-transform infrared spectroscopy (FT-IR)

The determination of the compatibility between gabapentin and the other constituents of the prepared GAB-SLNs was conducted using infrared (IR) spectroscopy. Individual mixtures of pure gabapentin, cholesterol, and GAB-SLN#2 were prepared by combining each substance with potassium bromide which was then compressed into discs (Unnisa et al., 2022). We used a Shimadzu 435 U-O4 IR spectrometer (Japan) to perform an IR scanning on each disc, covering 4,000 cm-1 - 400 cm-1 (Tian et al., 2018; Nasiri et al., 2020).

#### 2.1.10 The differential scanning calorimetry (DSC)

Thermal analysis is a method that can be used to determine the degree to which compounds crystallize. An empty aluminum pan served as the standard for this experiment. The experiment involved subjecting samples of pure gabapentin, cholesterol, and GAB-SLN#2 to a controlled heating process. The samples were put in an aluminum pan and subjected to gradual heating from 25°C–250°C (changed 10°C during every minute). Throughout the heating process, continuous flow of nitrogen gas at a rate of 20 mL per minute. was maintained (Gumireddy et al., 2019; Asif et al., 2022).

### 2.2 Pharmacological screening for the retinoprotective effect of gabapentin SLNs

#### 2.2.1 Experimental design

The animal protocol received approval by the ethical committee at the Faculty of Pharmacy in Suez Canal University (certificate code# 202302RA4 approved in February 2023). Thirty-six adult male Wistar rats were acclimatized to the housing conditions for 1 week under standard conditions of temperature and humidity. Thereafter, type 1 diabetes mellitus (T1DM) was experimentally induced using streptozotocin (STZ) solution in citrate buffer (pH = 4.5) (i.p., 30 mg/kg at day 1 and day 8) (Mohammad et al., 2023). To confirm the induction of T1DM, fasting blood glucose was assessed 72 h following STZ injection from a blood sample from the tail tip using a glucometer. T1DM was considered when the fasting blood glucose values exceeded 200 mg/dL.

Six weeks after diabetes confirmation, rats received drug treatments or vehicles via the oral route for 6 weeks.

Group I: The vehicle control group included healthy rats that received intraperitoneal doses of citrate buffer (instead of STZ).
Group II: DR control group,
Group III: DR + oral free gabapentin (10 mg/kg per day) (Reda et al., 2016),
Group IV: DR + oral free gabapentin (20 mg/kg per day).
Group V: DR + oral GAB-SLN#2 (10 mg/kg per day).
Group VI: DR + oral GAB-SLN#2 (20 mg/kg per day).

After finishing the experiment, rats were anesthetized by ketamine (80 mg/kg, i.p.) and killed by cervical dislocation. Then, retinas from the right and left eyes were dissected; the right retinas were kept in RIBA buffer and flash frozen for further homogenization and utilization for enzyme-linked immunoassays (ELISA) while the left retinas were fixed in 10% paraformaldehyde solution for further pathological studies.

#### 2.2.2 Assessment of retinal levels of inflammation and angiogenesis markers

The supernatants of retinal homogenates were aliquoted. ELISA method was used for the assessment of interleukin 6 (IL-6, MBS355410, My BioSource), Janus kinase (JAK, DL-JAK2-Ra, Jentaur, Kampenhout, Belgium), signal transducer and activator of transcription (STAT3, LS-F4933, LSBio, Seattle, WA 98121, USA) and VEGF (MBS2514825, MyBiosource) in retinal homogenate using commercially available kits. We read the color of the reaction product by an ELISA reader.

#### 2.2.3 Histopathological studies

After animal sacrification, the globe was dissected from orbit and fixed in paraformaldehyde. Then, the eyes were washed for half an hour and then placed in 60% ethanol for 120 min. Then the eyes were cut open from back to front. For preparing tissue cross-sections, we used serials of ethanol (70, 80, 95, and 100%). Then, eyes were embedded in paraffin, and 4–5 μm sections were cut at the level of the retina. These sections were subjected to staining with hematoxylin and eosin (H&E). The last step was mounting the slides and cover slipping before evaluation for morphopathological changes, imaging, and image analysis.

Further, other retinal sections were immunostained for VEGF. Microscopic fields were captured using a calibrated standard digital microscope camera (Tucsen ISH1000 digital microscope camera) using an Olympus^®^ CX21 microscope, with a resolution of 10 MP (megapixels) (3,656 × 2,740 pixels for each image) and we used “IS Capture” software for capture and image enhancements. All images were captured at ×400 original magnification (objective ×40) utilizing the UIS optical system (Universal Infinity System, Olympus^®^, Japan). Image analysis for the.

#### 2.2.4 Statistical analysis

The present data are presented as the mean ± standard deviation of the mean (SDM). The statistical software GraphPad Prism was utilized to assess the significance of differences between the study groups. This was achieved by doing a one-way ANOVA test, followed by a comparison between each pair of the study groups. Data were two-tailed and every probable comparison was highlighted. The statistical significance level is denoted as *p* < 0.05.

## 3 Results

Four GAB-SLNs were designed using a 2^2^ full factorial design and developed by the emulsification ultrasonication technique. The present study investigated the impact of lipid type and surfactant type on the following parameters: Y1 (EE%), Y2 (PS) (Y3) ZP, (Y4) PDI, and (Y5) R% of the produced formulations. Table 3 displays the outcomes of these variables.

**TABLE 3:** Dependent variables of GAB-SLNs prepared according to a 2^2^ factorial design.

Formula #	Y1: EE %	Y2: PS (nm)	Y3: ZP (mV)	Y4: PDI	Y5: R%
GAB-SLN #1	93.81 ± 2.38	213.58 ± 4.28	−29.37 ± 0.64	0.37 ± 0.05	96.46 ± 2.91
GAB-SLN #2	98.64 ± 1.97	185.65 ± 2.41	−32.18 ± 0.98	0.28 ± 0.02	99.27 ± 3.48
GAB-SLN #3	86.34 ± 2.12	320.51 ± 5.85	−22.45 ± 1.58	0.45 ± 0.01	89.19 ± 1.25
GAB-SLN #4	90.73 ± 1.88	288.47 ± 3.59	−24.89 ± 1.12	0.32 ± 0.01	92.34 ± 2.51

GAB-SLNs: gabapentin solid lipid nanoparticles.

### 3.1 The impact of the type of lipid and surfactant on the EE % (Y1)

The EE% refers to the proportion of drug entrapped and encapsulated within the nanoparticles that have been developed, relative to the overall quantity of drug provided. The EE% of the prepared GAB-SLNs exhibited a range of values from 86.34% ± 2.12% to 98.64% ± 1.97%, as shown in Table 3. According to the data presented in Figure 1 and Supplementary Table S1, it was observed that the EE% exhibited a statistically significant increase (*p* < 0.05) in formulations including cholesterol, whereas a decrease in EE% was observed in formulations containing stearic acid.

Furthermore, it was observed that the formulations including Pluronic F-68 exhibited an increment (*p* < 0.05) in the EE% compared to the formulations using Tween 80. The equation provided represents the connection between the independent variables and the EE% (Y1) in the study:

$$\text{EE}\%\;\left(Y1\right)=92.38+3.84\;X1+2.30\;X2$$

### 3.2 The effect of surfactant and lipid types on Y2 (PS), Y3 (ZP), and Y4 (PDI) of the GAB-SLNs

It was found that PS for GAB-SLNs ranged from 185.65 ± 2.41 nm for GAB-SLN #2 to 320.51 ± 5.85 nm for GAB-SLN #3 (Table 3). As shown in Figure 1 and Supplementary Table S2, the PS was significantly reduced by using cholesterol as a solid lipid. Additionally, the PS of GAB-SLNs was influenced by the specific surfactant employed. The study revealed a substantial decrease (*p* < 0.05) in PS when Pluronic F-68 was utilized in comparison to Tween 80. The equation provided represents the relationship between the independent variables and PS (Y2):

$$\text{PS }\left(Y2\right)=252.05-52.44\;X1-14.99\;X2$$

The ZP refers to the electric charge that is present at the border between nanoparticles and the surrounding media. The ZP is an indicator of the physical stability of the colloidal dispersion of SLNs (Das et al., 2011). The increase in the value of ZP, regardless of whether it is due to a positive or negative charge, leads to an increase in the repulsion between SLNs. Consequently, there is no aggregation between SLNs, resulting in enhanced stability (Das and Chaudhury, 2011).

The results indicate that the ZPs of all the produced GAB-SLNs were in range from −22.45 ± 1.58 mV to −32.18 ± 0.98 mV, as shown in Table 3. The findings from the 3D response plot (Figure 1) and ANOVA analysis (Supplementary Table S2) indicate that both the solid lipid type and surfactant type exerted a significant impact on ZP. This equation indicates the effect of independent variables in the ZP (Y3):

$$\text{ZP }\left(Y3\right)=-27.22-3.55\;X1-1.31\;X2$$

The PDI serves as an indicator of the uniformity of particle sizes within a given formulation. According to the data presented in Table 3, it was observed that the PDI values of GAB-SLNs formulas were below 0.5.

As shown in Figure 1 and Supplementary Table S2, the PDI value was reduced by using cholesterol as a solid lipid and Pluronic F-68 as a surfactant (Figure 1). The analysis of variance (ANOVA) indicated that neither the lipid type nor the surfactant type had a statistically significant impact on the PDI value (*p* > 0.05, Supplementary Table S2). The equation provided reflects the representation of the effect of independent variables in the PDI (Y4):

$$\text{PDI }\left(Y4\right)=0.3550-0.0300\;X1-0.0550\;X2$$

### 3.3 The impact of lipid and surfactant types on R% (Y5) of the prepared GAB-SLNs

**FIGURE 2:**
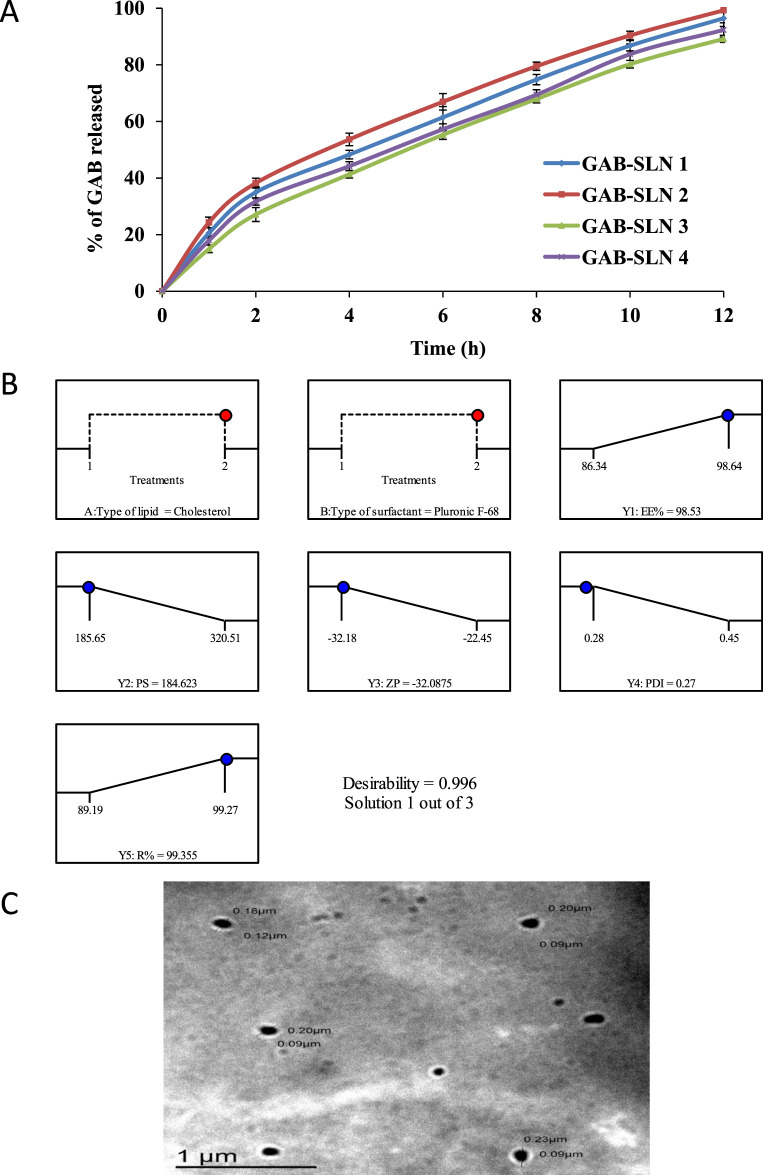
**(A)** The *in vitro* release pattern of gabapentin from GAB-SLNs. **(B)** The real and expected value of the optimal formulation (GAB-SLN#2), **(C)** The TEM image of the GAB-SLN#2.

The drug release rate is influenced by the composition of the prepared SLNs. According to the data shown in Table 3, the cumulative drug release from GAB-SLN formulations was observed to range from 89.19% ± 1.25% for GAB-SLN #3% to 99.27% ± 3.48% for GAB-SLN #2 after a duration of 12 h. The controlled release of gabapentin from the SLN formula was seen for 12 h, as depicted in Figure 2A.

The analysis of variance (ANOVA) conducted on the drug release % (Y5) data, demonstrated that both the lipid type and surfactant type (Supplementary Table S1) exerted a statistically significant influence on the drug release % values (*p* < 0.05). The following equation shows the influence of independent variables on the drug release % (Y5):

$$R\%\;\left(Y5\right)=94.31+3.55\;X1+1.49\;X2$$

The Higuchi diffusion model was the most appropriate mechanism for gabapentin release from the developed GAB-SLNs as determined by the greatest value of (r) (Table 4). Shazly conducted a study involving the development of ciprofloxacin SLNs and arrived at a similar conclusion. The findings indicated that the Higuchi diffusion model provided the most accurate fit for the release data (Shazly, 2017).

**TABLE 4:** The correlation coefficient (r) for the *in-vitro* release of gabapentin from the prepared GAB-SLNs using kinetic orders and systems.

Formulation	Correlation coefficients					
	0	1st	2nd	Diffusion	B-L	H-C
GAB-SLN #1	0.9937	−0.9480	0.7710	0.9977	0.9608	0.9846
GAB-SLN #2	0.9906	−0.8995	0.6718	0.9995	0.9586	0.9733
GAB-SLN #3	0.9947	−0.9827	0.8951	0.9981	0.9718	0.9958
GAB-SLN #4	0.9949	−0.9701	0.8574	0.9961	0.9644	0.9901

### 3.4 The optimization process for GAB-SLNs

The purpose of the optimization method was to choose the GAB-SLNs formulation that produced the best results with the dependent variables (responses) values that were specified. In terms of the dependent variables, the objective was to achieve the highest possible EE% (Y1), the lowest possible PS (Y2), the highest possible ZP (Y3), the lowest possible PDI (Y4), and the highest possible R% (Y5). To ascertain the most favorable value of the independent variables for achieving the desired responses, a 2^2^ factorial design was utilized. The GAB-SLN #2 was chosen as the optimal formula, which was made with a high level of X1 (+1, cholesterol), as well as a high level of X2 (+1, Pluronic F-68) (Figure 2B). The utilization of a 2^2^ factorial design proved to be effective in the identification of the optimized formulation, as evidenced by the proximity between the predicted and actual values, yielding a desirability index of 0.996.

### 3.5 Transmission electron microscopy of GAB-SLN #2

The TEM image of GAB-SLN #2, as depicted in Figure 2C, demonstrates that the nanoparticles produced exhibited a spherical and uniform morphology, devoid of any instances of aggregation.

### 3.6 Fourier transform infrared spectroscopy of GAB-SLNs

**FIGURE 3:**
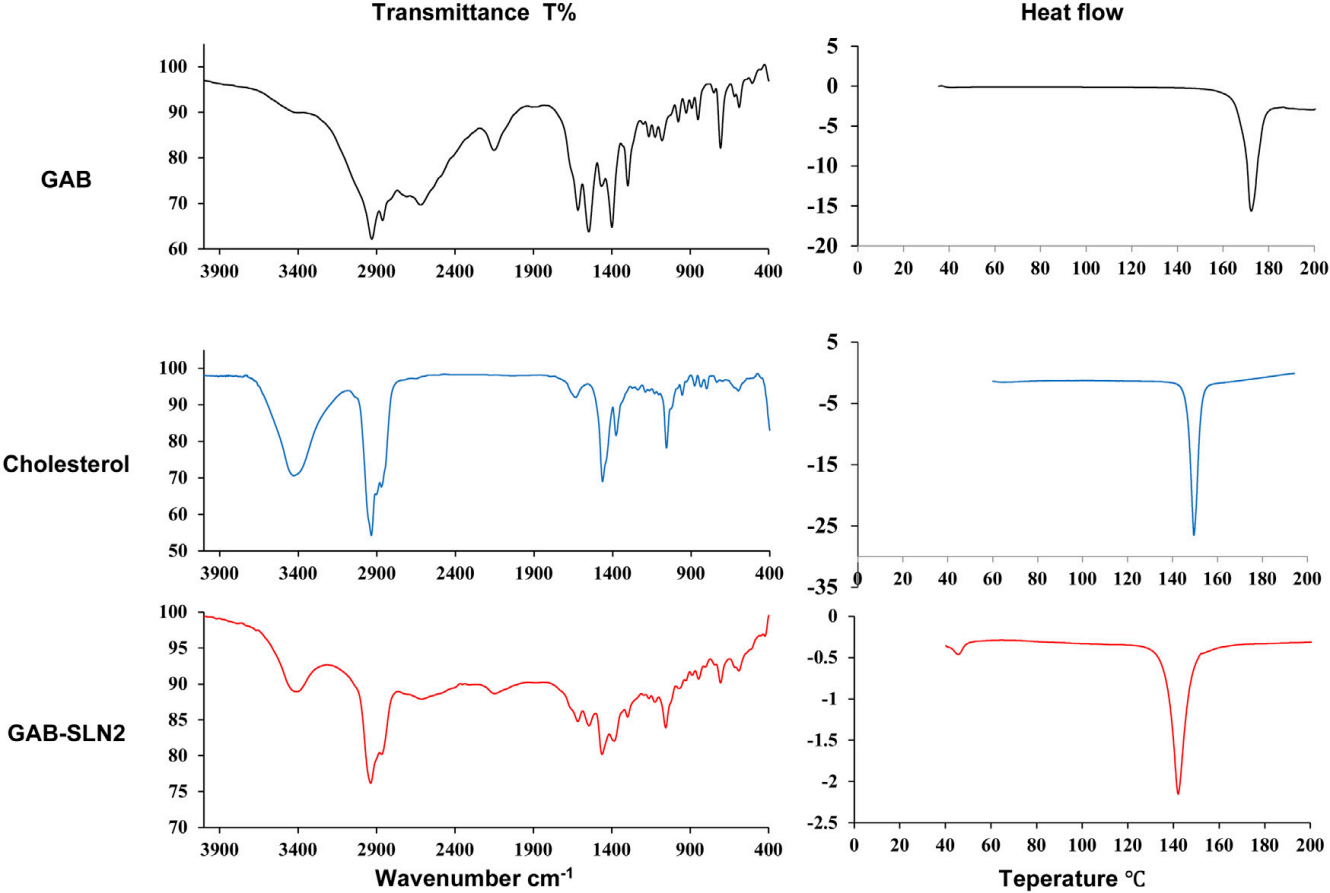
**(A)** Infrared spectroscopy and **(B)** DSC thermogram of gabapentin, cholesterol, and GAB-SLN #2.

The compatibility between the constituents in a pharmaceutical formulation can be determined by the utilization of infrared (IR) spectroscopy. The results of the IR spectroscopy performed on pure gabapentin, cholesterol, and GAB-SLN#2 are presented in Figure 3A.

The IR spectrum of gabapentin displayed several peaks indicative of certain molecular vibrations. These included the (amin N-H stretching) peak at 2,930 cm-1 and 2,860 cm-1, the (N-H) band at 2,150 cm-1 representing an additional vibrational band, the (carbonyl C=O) peak at 1,615 cm-1, the (C-O) band of carboxylate at 1,400 cm-1, and the (C-N stretching) peak at 1,163 cm-1. Choknud et al. came to the same conclusions since they revealed that the IR spectrum of GAB displayed the same peaks (Choknud et al., 2012).

The IR spectrum of cholesterol exhibited distinct peaks corresponding to specific functional groups. These peaks include the hydroxyl group (-OH) stretching at 3,430 cm-1, cyclic carbon-hydrogen (C-H) stretching at 3,035 cm-1, methyl carbon-hydrogen (C–H) stretching 3,000–2,800 cm−1 range, carbon-carbon (C=C) stretching at 1,464 cm−1, carbon-oxygen (C-O) stretching at 1,378 cm−1, in addition to a sharp peak at 1,057 cm−1, which can be attributed to ring deformation in cholesterol. Rostamkalaei et al. achieved similar outcomes, as they observed that the infrared spectrum of cholesterol exhibited identical distinctive peaks (Rostamkalaei et al., 2019). The IR spectra of GAB-SLN#2 exhibited distinct peaks corresponding to the distinctive vibrational frequencies of gabapentin and cholesterol.

### 3.7 The thermal analysis

Thermal analysis is a tool for determination of the physical change in the crystallinity of substances with heat. The DSC is one thermal analysis method widely used in the pharmaceutical field. In this work, the DSC analysis of pure gabapentin, cholesterol, and GAB-SLNs was done. The DSC thermogram of gabapentin, as shown in Figure 3B, demonstrated a sharp peak indicating endothermic behavior at 172°C, equivalent to its melting point. A similar finding was obtained previously (Hsu et al., 2010); the authors reported that the DSC thermogram of gabapentin exhibited an endothermic behavior at 171°C.

The DSC thermogram of cholesterol exhibited a peak indicating endothermic behavior at 149°C which corresponds to its melting point. This result was similar to that obtained by Rostamkalaei et al., who found that the DSC of cholesterol demonstrated a peak in the endothermic reaction at 150 C (Rostamkalaei et al., 2019). The DSC thermogram of GAB-SLN#2, which was formulated in the presence of cholesterol (solid lipid), showed one endothermic peak at 142°C which corresponds to cholesterol with small shit to lower temperature and decrease in the intensity as compared to the pure cholesterol, while the peak of gabapentin disappeared.

### 3.8 Results of the *in vivo* experiment

#### 3.8.1 GAB-SLN #2 downregulated IL-6/JAK/STAT3 proteins in retinas of diabetic rats

The present results indicated elevated levels of IL-6, JAK2, STAT3, and VEGF proteins in the diabetic rats compared to the vehicle-treated rats (Table 5). Treatment with the low dose of gabapentin (10 mg/kg) did not produce significant downregulation of these inflammatory proteins but the high dose of gabapentin (20 mg/kg) produced significant reductions in these proteins compared to the diabetic group. On the other hand, both doses of GAB-SLN#2 (10 or 20 mg/kg) reduced the retinal level of these proteins significantly; however, the higher dose produced a greater effect than the low dose (Table 5).

**TABLE 5:** Effect of GAB-SLN#2 on retinal level of IL-6, JAK2, STAT3, and VEGF.

Groups	IL-6 (pg/g)	JAK2 (ng/g)	STAT3 (ng/g)	VEGF (pg/g)
Vehicle	13.36 ± 2.045	2.65 ± 0.46	2.65 ± 0.49	9.6 ± 0.68
DR	50.3 ± 6.60*	13.35 ± 2.52*	10.39 ± 1.02*	31.98 ± 2.92*
DR + GAB (10 mg/kg)	42.97 ± 5.93*	11.27 ± 1.62*	8.57 ± 1.60*	26.4 ± 2.32*
DR + GAB (20 mg/kg)	32.85 ± 3.71^*$^	7.51 ± 0.58^*$^	6.60 ± 1.28^*$^	20.3 ± 1.95^*$^
DR + GAB-SLN#2 (10 mg/kg)	31.03 ± 3.71^*$^	7.43 ± 0.89^*$^	6.23 ± 0.96^*$^	21.23 ± 3.52^*$^
DR + GAB-SLN2# (20 mg/kg)	24.78 ± 1.73^$#@^	4.74 ± 0.78 ^$#@^	4.44 ± 0.58^$#@^	15.92 ± 0.92^$#@^

Data are mean ± SD. *: versus vehicle, $: versus DR, control, #: versus DR + gabapentin 10, @: versus DR + gabapentin 20, Ф: versus DR + GAB-SLN, 10, at *p* less than 0.05.

#### 3.8.2 GAB-SLNs improved retinal measurements in diabetic rats

**FIGURE 4:**
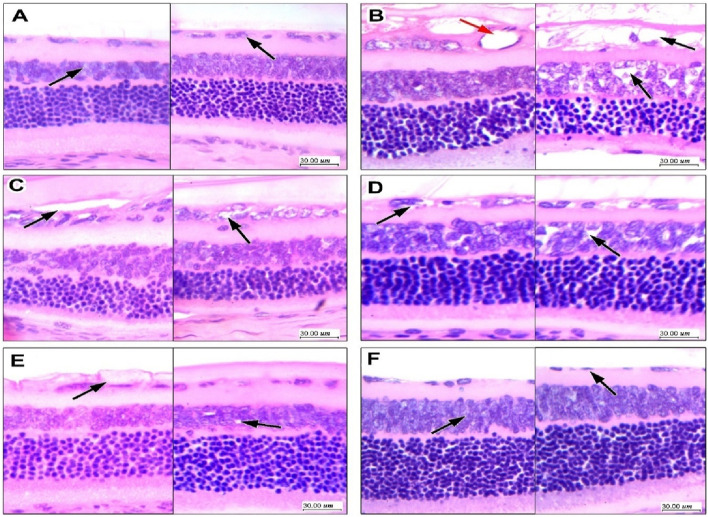
Effect of gabapentin on histopathologic features of diabetic retinopathy in retinal sections stained with hematoxylin and eosin. **(A)** Sections in the retina in the vehicle group show regular ganglion cell layers of single cells with inner and outer nuclear layers regularly arranged, with no vacuolation or disruption. **(B)** Sections from the DR control group showing marked vacuolar degeneration of ganglion cell layer with large cytoplasmic vacuoles and enlarged vesicular nuclei showing small nucleoli. There are visible dilated new vessels formed with thin endothelial cell lining (red arrow), there is also focal vacuolation of the inner nuclear layer with an irregular arrangement of cells. **(C)** The DR + gabapentin 10 mg/kg group showed moderate improvement; there is residual moderate vacuolar degeneration of ganglion cells layer with enlarged nuclei, and moderate vacuolation is also noted in INL and focally disrupting cells. **(D)** The DR + gabapentin 20 mg/kg group showed moderate improvement; there is residual moderate vacuolar degeneration of ganglion cells layer with enlarged nuclei, and moderate vacuolation is also noted in INL and focally disrupting cells. **(E)** The DR + GAB-SLNs 10 mg/kg group marked improvement with residual minimal vacuolation of ganglion cell layer with cells arranged in a single layer, few showed minimal nuclear enlargement, INL showed no changes, **(F)** The DR + GAB-SLNs 20 mg/kg group showing marked improvement with ganglion cell layer showing a single layer of cells with regular smaller nuclei and no vacuolar changes, no changes in INL or outer layers, no vessels visible in ganglion cells layer.

The H&E staining of retinas from vehicle-treated rats showed regular ganglion cell layers (GCL) of single cells with inner (INL) and outer nuclear layers (ONL) regularly arranged (Figure 4A) however, retinas from DR control rats showed marked vacuolar degeneration of GCL with large cytoplasmic vacuoles and enlarged vesicular nuclei showing small nucleoli in addition to dilated new vessels (Figure 4B). The DR + gabapentin (10 or 20 mg/kg) groups showed mild-moderate improvements in the histopathologic features and degeneration of GCL (Figures 4C,D). The DR + GAB-SLN2# (10 mg/kg) group showed moderate degeneration with residual minimal vacuolation of GCL with cells while few cells showed minimal nuclear enlargement (Figure 4E). The diabetic + GAB-SLN 2#(20 mg/kg) group showed improvement in GCL which appeared in a single regular layer of cells and without any noticed vacuolar changes. Further, the INL and ONL did not show pathologic changes or angiogenesis in the GCL (Figure 4F).

**FIGURE 5:**
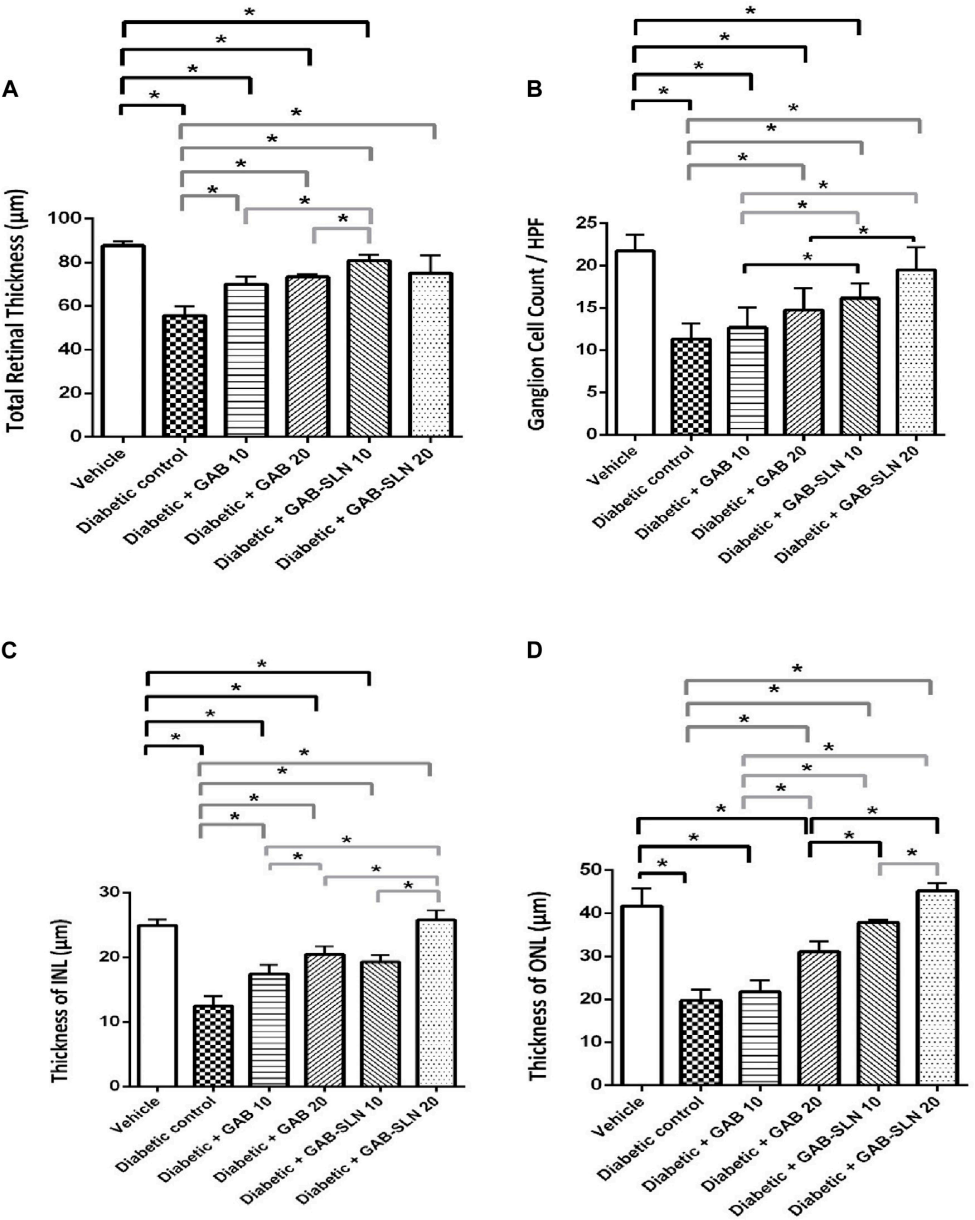
Measurements of retinal sections stained with hematoxylin and eosin. **(A)** Total retinal thickness, **(B)** retinal ganglion cell count/high power field (HPF), **(C)** Thickness of the INL, and **(D)** thickness of the ONL. Data are mean ± SD. *: at *p* less than 0.05.

The measurements of the retinal sections are shown in Figure 5. The total retinal thickness, ganglion cell count, INL thickness, and ONL thickness declined in diabetic rats compared to the vehicle-treated rats (Figures 5A–D). DR + GAB 10 or 20 mg/kg groups showed improvements in these measurements. Importantly, DR + GAB-SLNs (10 or 20 mg/kg) showed greater improvements in the measured parameters versus the DR + gabapentin (10 or 20 mg/kg) groups (Figures 5A–D).

**FIGURE 6:**
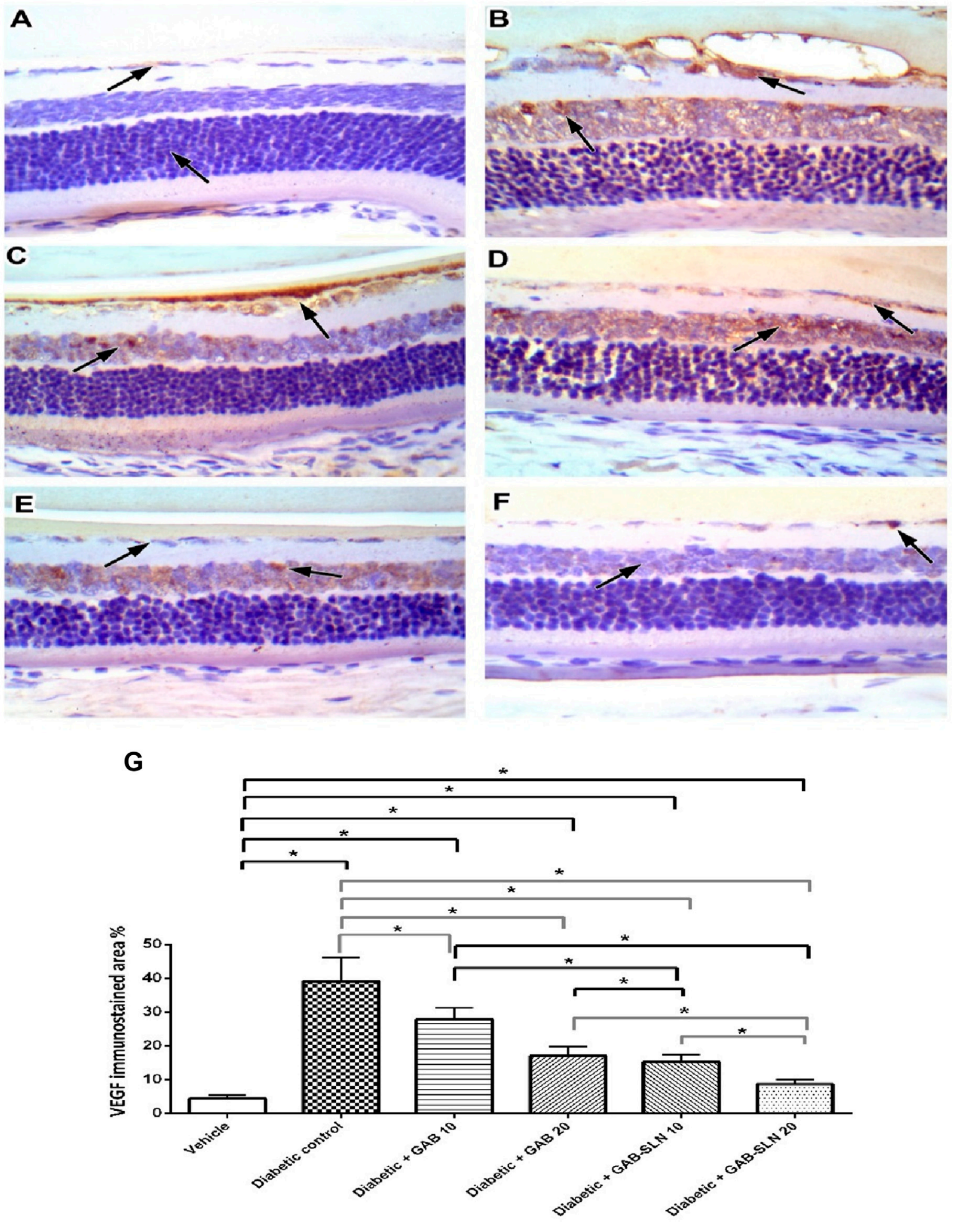
Measurements of retinal sections immunostained for VEGF. **(A)** The vehicle group, sections showed focal minimal weak staining in the inner nuclear layer with a negative ganglion cell layer. **(B)** the DR group shows moderate to strong staining of vascular structures lining cells, and moderate staining of vacuolated ganglion cell layer with moderate staining of the inner nuclear layer. **(C)** The DR + GAB 10 group shows moderate focal staining in the inner nuclear layer and moderate staining in the ganglion cell layer. **(D)** The DR + GAB 20 group shows weak staining in the ganglion cell layer with residual moderate staining in the inner nuclear layer. **(E)** The DR + GAB-SLN 10 mg/kg showed faint staining in the ganglion cell layer with mild staining in the inner nuclear layer. **(F)** The DR + GAB-SLN 20 mg/kg shows very focal weak staining of the inner nuclear and ganglion cell layer. **(G)** Column chart for mean immunostaining values. Data are mean ± SD. *: at *p* less than 0.05.

In Figure 6, immunostaining for VEGF is demonstrated in the study groups. The vehicle control group showed focal minimal weak staining in the INL with negative GCL (Figure 6A). The DR control group showed moderate to strong staining of vascular structures lining cells, and moderate staining of vacuolated GCL with moderate staining of the INL (Figure 6B). The DR + GAB 10 group shows moderate focal staining in the INL and moderate staining in the GCL (Figure 6C). The DR + GAB 20 group showed weak staining in the GCL with residual moderate staining in the INL. The DR + GAB-SLN 10 mg/kg group showed faint staining in the GCL with mild staining in the INL. The DR + GAB-SLN 20 mg/kg showed very focal weak staining of the INL and GCL. The comparison between the study groups is shown in Figure 6F. The DR control group revealed several fold increments in immunostaining for VEGF. Free gabapentin resulted in significant declines in VEGF immunostaining however GAB-SLNs decreased immunostaining for VEGF to a greater extent.

## 4 Discussion

Solid lipid nanoparticles are colloidal dispersion prepared by emulsification of solid lipid and aqueous phase in the presence of surfactant. The characteristics of SLNs are highly affected by their composition (lipid and surfactant type). The present study revealed a significant increase in the EE% when cholesterol and Pluronic F-68 were included in the formulations. Conversely, a decrease in EE% was observed in formulations including stearic acid and tween 80. These observations could perhaps be attributed to the disparity in molecular structure between cholesterol (C27 H46 O) and stearic acid (C18 H36 O2), specifically the longer carbon chain length of cholesterol. According to previous research, it has been observed that as the carbon chain length increases, the SLNs exhibit a decreased level of orderliness and an increased capacity for drug entrapment within the cavities (Shazly et al., 2018). The findings of this study align with the research conducted by Qushawy et al., which showed that the EE% of carbamazepine SLNs was greater when formulated with glyceryl monostearate compared to stearic acid. This difference was ascribed to the longer carbon chain present in glyceryl monostearate (Qushawy et al., 2019; Güney et al., 2014., prepared ascorbic acid, a hydrophilic drug, as solid lipid nanoparticles by hot homogenization method to improve the cellular uptake and anticancer activity. They found that EE% of ascorbic acid in SLNs above 90% evident that SLNs produced by the hot homogenization method could achieve a high drug incorporation (Güney et al., 2014).

Additionally, the observed outcomes could be linked to the elevated value of hydrophilic-lipophilic balance (HLB) of Pluronic F-68, which led to the formation of SLNs with enhanced stability and reduced drug leakage. Also, the utilization of a surfactant with a high HLB value may contribute to improved encapsulation of hydrophilic drugs (Bnyan et al., 2018). The findings were consistent with the results reported previously and documented an increase in the EE% of ibuprofen SLNs when a surfactant with a high HLB value was utilized (Bagde et al., 2019).

The PS was significantly decreased (*p* < 0.05) by using Pluronic F-68 as a surfactant and cholesterol as a solid lipid. These outcomes may be due to the classification of cholesterol as a surfactant due to the presence of hydrophilic and hydrophobic parts. Being a surfactant, cholesterol facilitated the emulsification process during SLN preparation resulting in a smaller size (Asasutjarit et al., 2007). The findings of this study align with those demonstrated a reduction in the size of SLNs upon the inclusion of cholesterol (Asasutjarit et al., 2007). The smaller size observed with Pluronic F-68 can be attributed to the greater HLB value, as reported in a previous study (Khames et al., 2019). Shahraeini et al. developed atorvastatin SLNs and observed a negative correlation between the particle size of the SLNs and the HLB value of the surfactant employed (Shahraeini et al., 2020).

The negative ZP seen can be ascribed to the ionization of the carboxylic group of gabapentin into carboxylate (COO-). The addition of cholesterol and Pluronic F-68 increased the value of ZP, whereas the inclusion of stearic acid and Tween 80 led to a decline in ZP value. The elevated negative charge observed in cholesterol can perhaps be linked to the presence of a hydroxyl group (-OH) on the cholesterol molecule, which bears a negative charge. The low value of PDI (<0.5) suggests a uniformity in the particle size distribution of all SLN formulations.

The cumulative drug release increased in formulations containing cholesterol and Pluronic F-68, but it was reduced in formulations containing stearic acid and Tween 80. The observations can be ascribed to the small size of the GAB-SLNs formulations that were developed with cholesterol and Pluronic F-68 (Venkateswarlu and Manjunath, 2004). The optimized formulation revealed the smoothness of the surface with no aggregation as shown by TEM. Prajapati et al. analyzed the surface morphology of isradipine SLNs in their study. The researchers observed that the SLNs displayed a spherical morphology and had a surface characterized by smoothness (Prajapati et al., 2018).

The *in vitro* release profile of GAB-SLNs exhibited two distinct phases. The initial phase, lasting for the first 2 h was characterized by rapid dissolution of gabapentin that was adsorbed on the surface of SLNs. This was followed by a sustained release phase, lasting for the subsequent 10 h when gabapentin was released through diffusion. Makoni et al. had a comparable outcome in their study, wherein they formulated efavirenz SLNs and observed a biphasic *in vitro* release profile (Makoni et al., 2019). According to the data presented in Figure 1, the cumulative drug release increased in formulations containing cholesterol and Pluronic F-68, but it was reduced in formulations containing stearic acid and Tween 80. The detected results can be attributed to the diminished size of the GAB-SLNs formulations that were developed with cholesterol and Pluronic F-68 (Venkateswarlu and Manjunath, 2004).

The TFIR spectroscopy observation indicates no evidence of any chemical interaction occurring between the drug and the other constituents present in the formulation. The results can be ascribed to the existence of all typical peaks of gabapentin in the IR spectrum of GAB-SLN #2. The lack of a gabapentin signal observed in the DSC thermogram of SLNs could be attributed to the drug being entrapped within the lipid matrix and existing in an amorphous state.

Inflammation is a hallmark of DM and dysregulated levels of inflammatory mediators precede both retinal neurodegeneration and microvascular damage (Tang and Kern, 2011; Alomar et al., 2021b). Various clinical and experimental studies reported increased levels of IL-6, a key pro-inflammatory cytokine, in DM (Canataroglu et al., 2005; Kawashima et al., 2007; Said et al., 2018; Xia et al., 2021). Indeed, IL-6 is one of the major cytokines crucially implicated in the pathogenesis of DM (Simó et al., 2018). Persistent increased elevation of proinflammatory cytokines results in chronic inflammatory status in the diabetic retina leading ultimately to leukocyte activation, leukostasis, and disruption blood-retinal barrier disruption (Hernández et al., 2019). Specifically, IL-6 demonstrated angiogenic effects on the vascular endothelial cells (Nilsson et al., 2005). Further, accumulated evidence reported IL-6 as a stimulus for retinal JAK-STAT3 signaling. Alsaffar et al. reported that IL-6 induced prolonged vascular permeability via JAK/STAT signaling pathway (Alsaffar et al., 2018). Additionally, activation of the JAK/STAT signaling pathway using IL-6 and other proinflammatory cytokines triggers the angiogenesis process mainly via VEGF activation (Xu et al., 2005). Also, triggering JAK/STAT3 signals accentuate growth factor and cytokine-induced angiogenesis in oxygen-induced retinopathy (Stahl et al., 2012). Moshapa et al. concluded that the proinflammatory IL-6/JAK/STAT axis participates in vascular disruption in type 2 diabetes mellitus (Moshapa et al., 2019). Interestingly, Ye and Steinle showed that suppression of the IL-6-mediated STAT3/VEGF pathway inhibited inflammation and apoptosis in retinal endothelial cells under hyperglycemic conditions (Ye and Steinle, 2017). Thereby, the IL-6/JAK/STAT3 axis plays a pivotal role in pathological angiogenesis of the retina under diabetic conditions and could be targeted for therapeutic management of DM. Previously, topical application of gabapentin downregulated IL-6 in experimental uveitis (Anfuso et al., 2017) giving credence to our findings. In agreement, several experimental studies demonstrated that inflammatory burdens were observed in diabetic rats (Ali et al., 2019) and mice (Zaitone et al., 2020).

In the current study, H&E staining was used to quantify the pathology in the retinal neurons. Many previous studies used H&E staining for exploring pathology in the CNS neurons (Elsherbiny et al., 2019b; El-Sherbeeny et al., 2020; Abdel-Sattar et al., 2021; Alomar et al., 2021a; Ateyya et al., 2024). In our hands, pathological features of DM were documented in the form of shrinkage of the total retinal thickness in diabetic rats and shrinkage in INL and ONL as well as a decline in GCL count. In agreement, some previous experimental studies documented similar pathology in the diabetic retinas (Elsherbiny et al., 2019a; ElSayed et al., 2023; Alshaman et al., 2024). Indeed, one of the limitations of this study is the lack of a group treated with standard medication like corticosteroids. This may be considered in future studies.

## 5 Conclusion

In conclusion, the present study demonstrated that preparing gabapentin in SLNs improved its physical properties. Four preparations were formulated and synthesized and the best formula (GAB-SLN#2) was selected for testing the biological activity as an anti-inflammatory neuroprotectant. On the other hand, oral doses of GAB-SLNs as well as free gabapentin protected against DR in experimental diabetic rats and mitigated the pathological features observed in the retinas. The protective efficacy of oral gabapentin was mediated, at least partly, through the suppression of IL-6/JAK2/STAT3 along with the reduction of VEFG levels in the diabetic retina. The superior effect of GAB-SLNs recommends preparing gabapentin in SLNs to improve its oral activity and to be studied further in other models of neurologic disorders.

In future studies, pharmacokinetic data would help understand how GAB-SLNs are more effective than gabapentin treatment alone. If a clinical study is planned, evidence should be provided to demonstrate superior efficacy or better risk-benefit balance for GAB and then GAB-SLNs over the standard of care.

## Data Availability

The raw data supporting the conclusions of this article will be made available by the authors, without undue reservation.
