# Supplementary material for: Formulation and characterization of cholesterol-based nanoparticles of gabapentin protecting from retinal injury
Source: Front Chem. 2024 Oct 21;12:1449380. doi: 10.3389/fchem.2024.1449380 (PMC11537204; doi:10.3389/fchem.2024.1449380)
Supplement: Supplementary file 1 [file DataSheet2.docx]

### Supplementary Table 2. Analysis of variance (ANOVA) for EE% (Y1), PS (Y2), ZP (Y3), PDI (Y4), and R% (Y5) of the synthesized GAB-SLNs.

| \| **Y1: EE%** \| \| \| \| \| \| \| \| \| --- \| --- \| --- \| --- \| --- \| --- \| --- \| --- \| \| **Source** \| **SS** \| **DF** \| **MS** \| \| **F value** \| ***P* value** \| \| \| **Model** \| 80.39 \| 2 \| 40.19 \| \| 830.46 \| 0.0245 \| \| \| A-X1 \| 59.14 \| 1 \| 59.14 \| \| 1221.82 \| 0.0182 \| \| \| B-X2 \| 21.25 \| 1 \| 21.25 \| \| 439.09 \| 0.0304 \| \| \| **Residual** \| 0.0484 \| 1 \| 0.0484 \| \|  \|  \| \| \| **Cor Total** \| 80.44 \| 3 \|  \|  \| \| \|  \| |
| --- | --- | --- | --- | --- | --- | --- | --- | --- | --- | --- | --- | --- | --- | --- | --- | --- | --- | --- | --- | --- | --- | --- | --- | --- | --- | --- | --- | --- | --- | --- | --- | --- | --- | --- | --- | --- | --- | --- | --- | --- | --- | --- | --- | --- | --- | --- | --- | --- | --- | --- | --- | --- | --- | --- | --- | --- |
| \| **Y2: PS** \| \| \| \| \| \| \| \| \| --- \| --- \| --- \| --- \| --- \| --- \| --- \| --- \| \| **Source** \| **SS** \| **DF** \| **MS** \| \| **F value** \| ***p* value** \| \| \| **Model** \| 11897.87 \| 2 \| 5948.93 \| \| 1408.69 \| 0.0188 \| \| \| A-X1 \| 10998.77 \| 1 \| 10998.77 \| \| 2604.48 \| 0.0125 \| \| \| B-X2 \| 899.10 \| 1 \| 899.10 \| \| 212.90 \| 0.0436 \| \| \| **Residual** \| 4.22 \| 1 \| 4.22 \| \|  \|  \| \| \| **Cor Total** \| 11902.09 \| 3 \|  \|  \| \| \|  \| |
| \| **Y3: ZP** \| \| \| \| \| \| \| \| \| --- \| --- \| --- \| --- \| --- \| --- \| --- \| --- \| \| **Source** \| **SS** \| **DF** \| **MS** \| \| **F value** \| ***P* value** \| \| \| **Model** \| 57.37 \| 2 \| 28.69 \| \| 838.15 \| 0.0244 \| \| \| A-X1 \| 50.48 \| 1 \| 50.48 \| \| 1474.98 \| 0.0166 \| \| \| B-X2 \| 6.89 \| 1 \| 6.89 \| \| 201.33 \| 0.0448 \| \| \| **Residual** \| 0.0342 \| 1 \| 0.0342 \| \|  \|  \| \| \| **Cor Total** \| 57.41 \| 3 \|  \|  \| \| \|  \| |
| \| **Y4: PDI** \| \| \| \| \| \| \| \| \| --- \| --- \| --- \| --- \| --- \| --- \| --- \| --- \| \| **Source** \| **SS** \| **DF** \| **MS** \| \| **F value** \| ***P* value** \| \| \| **Model** \| 0.0157 \| 2 \| 0.0078 \| \| 19.62 \| 0.1576 \| \| \| A-X1 \| 0.0036 \| 1 \| 0.0036 \| \| 9.00 \| 0.2048 \| \| \| B-X2 \| 0.0121 \| 1 \| 0.0121 \| \| 30.25 \| 0.1145 \| \| \| **Residual** \| 0.0004 \| 1 \| 0.0004 \| \|  \|  \| \| \| **Cor Total** \| 0.0161 \| 3 \|  \|  \| \| \|  \| |
| \| **Y5: R%** \| \| \| \| \| \| \| \| \| --- \| --- \| --- \| --- \| --- \| --- \| --- \| --- \| \| **Source** \| **SS** \| **DF** \| **MS** \| \| **F value** \| ***P* value** \| \| \| **Model** \| 59.29 \| 2 \| 29.65 \| \| 1025.79 \| 0.0221 \| \| \| A-X1 \| 50.41 \| 1 \| 50.41 \| \| 1744.29 \| 0.0152 \| \| \| B-X2 \| 8.88 \| 1 \| 8.88 \| \| 307.28 \| 0.0363 \| \| \| **Residual** \| 0.0289 \| 1 \| 0.0289 \| \|  \|  \| \| \| **Cor Total** \| 59.32 \| 3 \|  \|  \| \| \|  \| |

SS: Sum Squares, MS: mean square
