# Supplementary material for: Formulation and characterization of cholesterol-based nanoparticles of gabapentin protecting from retinal injury
Source: Front Chem. 2024 Oct 21;12:1449380. doi: 10.3389/fchem.2024.1449380 (PMC11537204; doi:10.3389/fchem.2024.1449380)
Supplement: Supplementary file 2 [file DataSheet1.docx]

Supplementary Table 1. The calibration curve for gabapentin was done in a phosphate buffer pH of 7.4. The linearity was in concentration 10-100 mcg/mL

|  | |
| --- | --- |
| conc (mcg) | Absorpance |
| 100 | 0.86 |
| 90 | 0.779 |
| 80 | 0.679 |
| 70 | 0.589 |
| 60 | 0.508 |
| 50 | 0.41 |
| 40 | 0.348 |
| 30 | 0.248 |
| 20 | 0.162 |
| 10 | 0.09 |


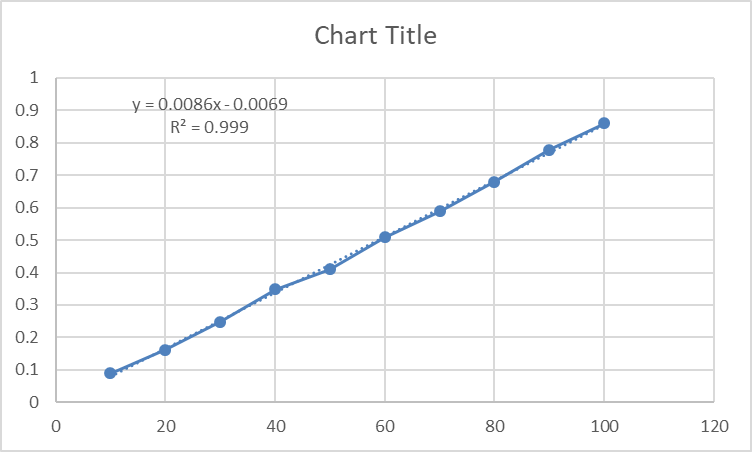


Supplementary Figure 1. Calibration curve for gabapentin.
